# Skin-impedance in Fabry Disease: A prospective, controlled, non-randomized clinical study

**DOI:** 10.1186/1471-2377-8-41

**Published:** 2008-11-06

**Authors:** Surya N Gupta, Markus Ries, Gary J Murray, Jane M Quirk, Roscoe O Brady, Jeffrey R Lidicker, Raphael Schiffmann, David F Moore

**Affiliations:** 1 Division of Pediatric Neurology, Department of Pediatrics, Penn State University College of Medicine, 500 University Drive, Hershey, Pennsylvania 17033, USA; 2 Developmental and Metabolic Neurology Branch, National Institute of Neurological Disorders and Stroke, National Institutes of Health, Bethesda, Maryland, USA; 3 Temple University Center for Statistical and Information Science, Temple University School of Medicine, Philadelphia, Pennsylvania, USA; 4 Section of Neurology, Department of Internal Medicine, University of Manitoba, Winnipeg, Manitoba, Canada; 5 Shire Human Genetic Therapies, Cambridge, MA, USA; 6 Institute of Metabolic Disease, Baylor Research Institute, 3812 Elm Street, Dallas, TX 75226, USA

## Abstract

**Background:**

We previously demonstrated improved sweating after enzyme replacement therapy (ERT) in Fabry disease using the thermo-regularity sweat and quantitative sudomotor axon reflex tests. Skin-impedance, a measure skin-moisture (sweating), has been used in the clinical evaluation of burns and pressure ulcers using the portable dynamic dermal impedance monitor (DDIM) system.

**Methods:**

We compared skin impedance measurements in hemizygous patients with Fabry disease (22 post 3-years of bi-weekly ERT and 5 ERT naive) and 22 healthy controls. Force compensated skin-moisture values were used for statistical analysis. Outcome measures included 1) moisture reading of the 100^th ^repetitive reading, 2) rate of change, 3) average of 60–110^th ^reading and 4) overall average of all readings.

**Results:**

All outcome measures showed a significant difference in skin-moisture between Fabry patients and control subjects (p < 0.0001). There was no difference between Fabry patients on ERT and patients naïve to ERT. Increased skin-impedance values for the four skin-impedance outcome measures were found in a small number of dermatome test-sites two days post-enzyme infusions.

**Conclusion:**

The instrument portability, ease of its use, a relatively short time required for the assessment, and the fact that DDIM system was able to detect the difference in skin-moisture renders the instrument a useful clinical tool.

## Background

Fabry disease is an X-linked lysosomal disorder caused by a deficiency of alpha-galactosidase A (GALA) resulting in accumulation of alpha-D-galactosyl conjugates, particularly globotriosylceramide in a variety of cell types [1,2], including dorsal root ganglia [3]. The diagnosis of Fabry disease is often made on clinical suspicion because of the presence of skin lesions (angiokeratoma) or corneal opacities that do not affect visual acuity. Other methods of diagnosis including a family pedigree ascertainment taking into account a history of renal failure or stroke of early onset, i.e. less than 50 years of age [4]. The demonstration of deficient GALA enzyme activity in white blood cells of hemizygous males or identification of a pathognomonic mutation in a heterozygous female confirms the diagnosis [5,6]. Neuropathic pain and hypohidrosis may also contribute to reduced exercise capacity and heat intolerance [7].

Extensive lipid accumulation within neurons of the autonomic nervous system may be the pathologic basis for neuropathic pain, diminished sweating, and other autonomic signs of this disorder. Enzyme replacement therapy (ERT) for Fabry disease has shown to be promising for adult hemizygous patients in the context of stabilizing renal disease and reversing less life-threatening symptoms such diarrhea [8,9].

We previously demonstrated improved sweating in adult male Fabry patients treated with ERT using thermo-regularity sweat testing (TST) and quantitative sudomotor axon reflex test (QSART) [10]. Both of these tests are difficult to implement in a routine clinical setting [11-13]. A more convenient clinical instrument allowing estimation of skin moisture uses dynamic dermal impedance (DDIM). "Impedance" is the resistance to the flow of an alternating current and is influenced by skin-moisture. The DDIM system is a portable instrument with force compensation to allow measurement of skin-moisture. DDIM has been used in the clinical setting to assess skin-moisture [14,15], but never in Fabry disease.

Here, we report the ascertainment of skin moisture across surface dermatomes by DDIM in patients with Fabry disease compared to healthy control subjects [15-17].

## Methods

### Subjects

Twenty-two adult male patients with Fabry disease who were after 3 years of ERT (mean age: 40.6 ± 8.1 years), five ERT-naïve patients with Fabry disease (mean age: 33.8 ± 8.0 years), and 22 control subjects (mean age: 36.0 ± 14.1 years) participated in this study. All but two were non-Hispanic Caucasians. Fabry disease was confirmed by GALA enzyme assays. The clinical manifestations of patients on ERT have been described previously in detail [18]. All the patients in this study had neuropathic pain and hypohidrosis.

The healthy control subjects were recruited through the National Institutes of Health Clinical Research Volunteer Program. They were interviewed and examined by the investigator and were confirmed as clinically and neurologically normal.

The Institutional Review Board of the National Institute of Neurological Disorders and Stroke at the National Institutes of Health approved this study. All patients and healthy control subjects gave their written informed consent prior to their inclusion in this study.

### GALA enzyme: production and treatment regimen

The α-galactosidase A enzyme used in this study (agalsidase alfa, Replagal^®^) was produced in a genetically engineered continuous human cell line (Shire Human Genetic Therapies, Cambridge, MA, USA). It was administered at a dose of 0.2 mg/kg of body weight by intravenous infusion over a period of 40 minutes at a frequency of every other week [18]. Patients who were on ERT for three years were studied just before enzyme infusion (defined as pre-infusion) and two days after enzyme infusion (defined as post-infusion).

### Dynamic dermal impedance monitor (DDIM) (Petite)

The Petite system DPM 9003 (NOVA Technology Corporation, Portsmouth, NH, USA) was used to assess the skin-impedance as a surrogate for skin moisture. [19].

### Testing and data collection

Fabry patients on ERT (22 patients) and ERT-naïve (5 patients) were studied twice (before and two days after their ERT infusion or two days after the initial measurement for those who were not on ERT). Skin moisture of healthy controls was measured only once. All tests were performed in the same room with controlled temperature and humidity. Temperature and humidity were recorded throughout the testing.

**Figure 1:**
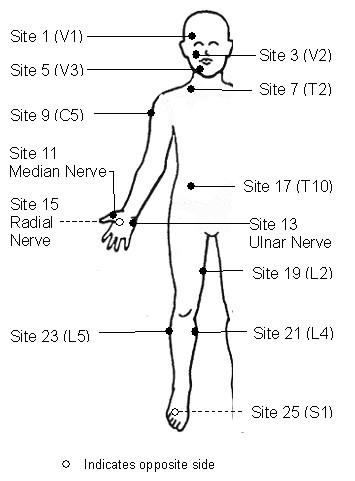
**Neuro-anatomical dermal test sites used for the skin-impedance testing.** The PDA probes were placed on specific locations on the body as marked by the bullets in the figure. Note the corresponding test sites are not shown on the left side of the body. The letter and number within the bracket indicate the specific cutaneous nerve distribution.

The hand-held DDIM probe connected to a personal digital assistant (PDA) was placed in a specific sequence on test-sites 1 through 26, corresponding to the cutaneous dermatomes from face to feet as shown in Figure 1. The recorded data were transferred from the PDA to a personal computer for subsequent analysis.

For each of 26 test-sites, the DDIM System recorded and displayed five columns of data; Column 1 – Time increment (five readings per second), Column 2 – Force-compensated moisture value (Dermal Phase Meter, DPM), Column 3 – Force applied (in grams), Column 4 – Skin surface temperature (in Celsius), Column 5 – Non-force-compensated moisture value. All five values were provided for approximately 0 – 120 readings. Only Column 2 data of force-compensated moisture values, for 1st–110^th ^recordings, at the 26 sites of each subject were used for statistical analysis. The parameter 'force-compensated moisture' was chosen because it provides a uniform application of the skin impedance probe from subject to subject and within a subject. The probe force compensation provides increased uniformity of measurement and therefore a reduced variance of the measurements.

**Figure 2:**
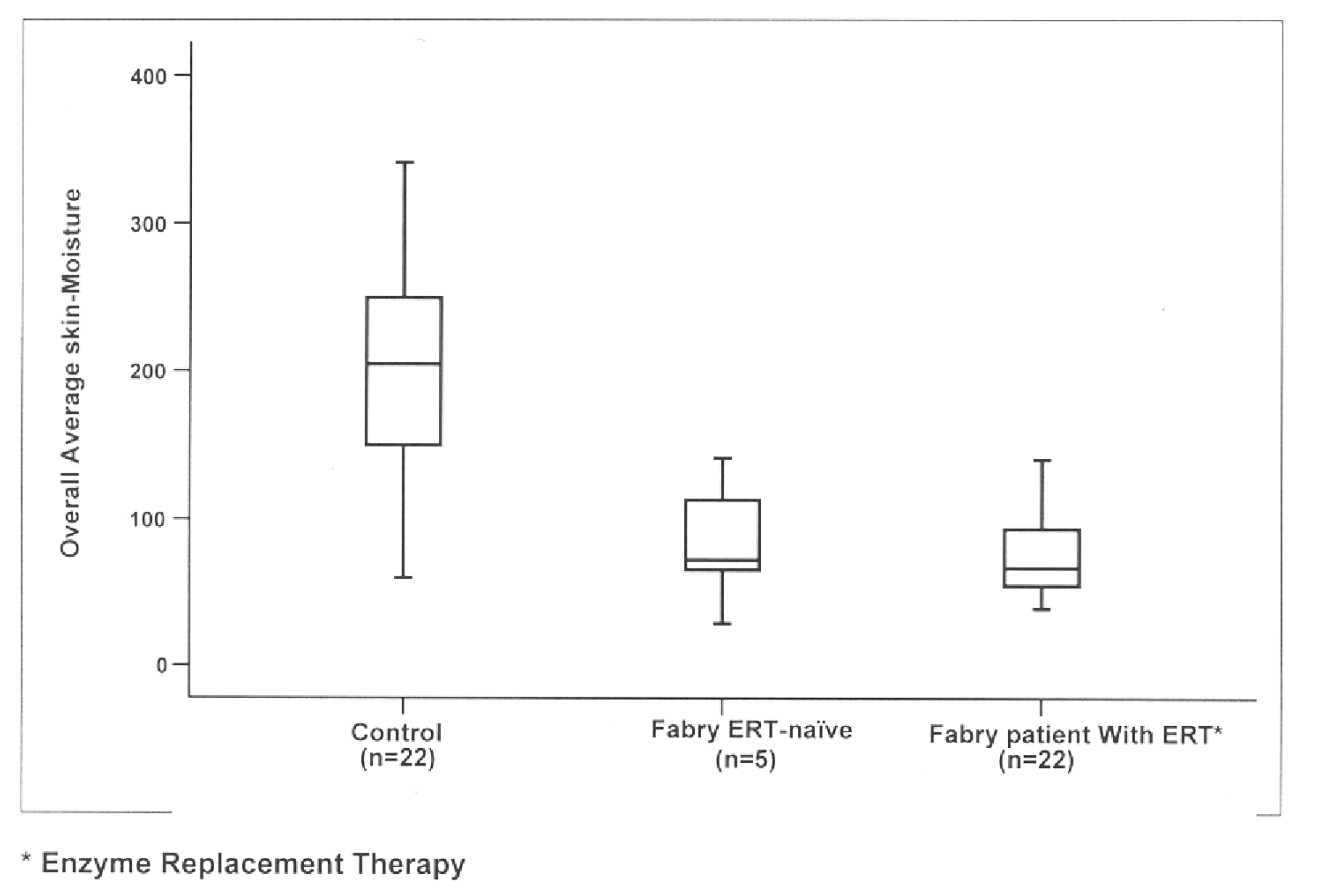
**This box plot of an Overall Average skin-moisture value in DPM units at right ophthalmic nerve distribution (test site 1) for three Groups of subject shows the difference in skin-moisture values between healthy controls and Fabry patients.** No difference in skin-moisture values between Fabry ERT-naïve and Fabry patient on enzyme replacement therapy was discerned.

A typical skin-impedance measurement value graph averaged across all 26 sites for Control group (n = 22) is shown in Figure 2.

### Creation of outcome measures

Four outcome measures were created to analyze various aspects of the force-compensated impedance values. The designs of the outcomes were intended to account for fact that there were 110 readings for each patient at each of 26 test-sites and to account for the distribution of the readings over time in four different ways (see Figure 2). The definition of each outcome measures is as follows:

1). Reading 100 (R100): The moisture reading of the 100^th ^of the 110 readings. This measure is designed to capture the extreme time values of the readings.

2). Rate of Change: Generally the moisture measurements increased from the 1^st ^to the 110^th ^for each reading. The rate of change or chord-slope of this increase was captured by taking the average of the first 10 readings (R = 1, 2...10) and subtracting it from the average of the last 10 readings (R = 100,102...110). Averages were taken at the beginning and end of the sample to make the measure resistant to outliers and large variations.

3). Average 60+: Of the 110 readings the 1^st ^through 59 readings were ignored and the rest (60–110) were averaged.

4). Overall Average: All 110 readings were combined into the mean.

### Statistical Analysis

SPSS version 14 (SPSS Inc, Chicago, IL, USA) was used for all analysis. Comparisons between groups were performed through ANOVA with Tukey post-hoc multiple comparisons. Tests using control variables for adjustment of differences were done using Generalized Linear Models. Tests of pre-versus post-ERT were performed using the paired two-sample t-test.

### Testing Environment

All subjects had normal skin temperature. Although the total difference in temperatures for the pre-infusion between the 3 subject groups (controls, pre-ERT-naïve and Fabry on ERT) differed by a maximum of 1.3°F, this small difference was statistically significant (*p *= 0.019). A similar situation occurred for pre-infusion humidity, post-ERT temperature, and post-infusion humidity (*p *= 0.043, *p *= 0.015, and *p *= 0.006, respectively).

## Results

All created outcomes, (R100, Rate of Change, Average 60+ and Overall Average), showed similar significant differences (p < 0.0001) in skin-impedance values between Fabry patients and control subjects for most test-sites. Based on p-values, sites 19 through 24 provided inconsistently small p-values in all four measures,, while sites 25 and 26 did not provide useful data for the Rate of Change parameter.

As a representative example, a box plot of the Overall Average moisture value at test-site 1 for three groups of subjects is shown in Figure 2. These differences persisted in the other three outcome measures (R100, Rate of Change and Average 60+), even after controlling for age, pre-infusion temperature differences and pre-infusion humidity differences as covariates, (*p *< 0.0001).

Since the control group is statistically independent of the four outcome measures, we used a simple ANOVA analysis incorporating all the sites and a Tukey Post-hoc analysis to show that no significant difference appeared between Fabry on ERT and Fabry naïve to ERT patients groups (Table 1).

**Table 1:** Tukey Post-hoc Results for Between Group Differences for one of the four created Overall Average skin-moisture outcome measures value at test-site 1, (right ophthalmic cutaneous nerve distribution)

Patient Group (I)	Patient Group (J)	Mean Difference (I-J)	Std. Error	Sig.
Control	Fabry ERT^1^-naïve	128.455(*)	37.638	0.004
	Fabry with ERT	132.636(*)	22.906	0.000
				
ERT-naïve Fabry	Control	-128.455(*)	37.638	0.004
	Fabry with ERT	4.182	37.638	0.993
				
Fabry with ERT	Control	-132.636(*)	22.906	0.000
	Fabry ERT-naïve	-4.182	37.638	0.993

Groups listed in "I" are compared to each in "J" and a significantly different mean is determined by the *p*-value shown in the "Sig" column.

^1^Enzyme Replacement Therapy

(*) Significant at 0.01 levels

### GALA Enzyme Models

There is a strong relation between the enzyme levels and group membership (p < 0.0001). Therefore, to avoid co-linearity problems with the model, we could not put both patient group and residual enzyme levels in the same model. Interestingly, when this attempt was made, enzyme level significantly predicted skin-impedance levels and patient group did not (p = 0.048, p = 0.52 respectively for Overall Average at Site 1). This indicates that enzyme levels predict skin moisture levels better than the patient groups. With enzyme levels replacing patient group in the model (removed patient groups), enzyme levels were significantly related to skin moisture values even after controlling for temperature and humidity (p < 0.0001).

### Pre-versus Post-ERT testing Skin-moisture values

Analysis of pre-infusion and post-infusion skin-impedance demonstrated no significant fixed effect attributable to ERT. A paired two-sample t-test was performed comparing pre-infusion and post-infusion scores at each site for each of the four created outcome measures. Test-site 2 (Left V1) is the only site that showed a significant difference between pre- and post-infusion moisture. However, this was only true for T-100 (*p *= 0.029), Average 60+ (*p *= 0.022), and Overall Average (*p *= 0.024), but not Rate of Change (*p *= 0.383). The matched site, Site1 (Right V1), was not significant for any of the outcome measures. The test-site 19 (Right L2) was significantly different in only the Rate of Change case with a decrease in skin-impedance. Recall that Site 19 also was the poorest performer otherwise.

## Discussion

The observed difference in skin-moisture values between healthy controls and Fabry patients is not surprising but it is important to note that the DDIM system was able to detect such a difference. In comparison with QSART, in which sweat function improved 24–72 hours after enzyme infusion, normalized in four patients and seven patients remained non-responders [10]. The DDIM system did not show improvements either 2 weeks after infusion, or 2 days after infusion in Fabry patients. The small sample size may explain the lack of difference in skin-moisture values between ERT-naïve patients and Fabry patients on ERT which may have implications for the use of skin-impedance in clinically monitoring ERT efficacy. Since all Fabry patients on ERT had fortnightly enzyme infusions over three years prior to this study, it seems that ERT does not have a cumulative effect on skin-impedance if one assumes that the pre-treatment values of the treated group were similar to the five ERT naïve patients. This is particularly obvious in pre- and two days post-ERT testing in Fabry patients.

Since each of the created outcome measures showed no particular performance advantage over the R100 outcome measure, we recommend the R100 measure due to its convenience in the clinical setting. Use of the R100 outcome requires that the user of the DDIM System need only needs to record a single skin-moisture reading (100^th ^reading of 110). This avoids having to use complicated database software and hours of programming to format the text data into summary information required for each of the other measures. However, it would be necessary to prospectively validate this index.

### Specific Dermal Test-site

In an analysis of test-sites, only a few sites performed poorly with R100 (sites 19 through 24). So, the investigator can choose any of the test-sites from 1–23 as a test-site on the subject body. We recommend the three most accessible dermatomes as test sites for use in Fabry disease; Site 1 (Face, right V1), Site 7 (Neck, right T2) and Site 17 (Hand, right radial). The median value from these three readings sites could be taken as an representative value of the overall skin impedance in a given patient in order to reduce the measurement variability

Our results of skin-moisture values between Fabry patients and control subjects are consistent with previous studies of TST and QSART in Fabry disease [10,11]. But unlike previous studies, this study failed to show an acute improvement in skin-moisture in post-ERT Fabry patients who had received ERT over three years periodically. This could be due to fact that the DDIM system, unlike previous studies, assesses the base level of skin-moisture and not thermal or chemical induced skin-moisture (sweating). The importance of measuring either base or induced skin-moisture is not known but may be explored. Because the therapeutic effect of ERT (assessed as a function of sweating) lasted for about 7 days with subsequent return to pre-infusion response level in previous studies, the ascertainment of post-infusion skin-moisture response in the current study may be improved by recording an induced skin-moisture response over an extended period.

### GALA enzyme and skin-moisture

The statistical relationship of residual GALA enzyme levels to skin-moisture in the analysis model value further corroborates a known association of hidrosis and residual GALA enzyme activity in Fabry disease. Patients with residual enzyme activity have milder disease, and normal or close to normal sweating [20]. Future study should explore whether an improvement in skin-impedance translates to improvement in other pathophysiological functions [21,22].

Although there was a statistically significant difference in environmental parameters by subjects group, the skin-impedance outcome measures were not affected. This was most likely due to the small magnitude of the environmental differences. However, efforts should be made to assure an adequate control of temperature and humidity in the testing environment in subsequent studies.

### Limitations of the study

The main limitation of this study was the lack of randomization and a lack of serial post-infusion testing. Although the DDIM instrument is designed to adjust for the pressure applied on skin surface and there is a built in indicator for a lack of proper skin contact, there was still evidence of significant variance in skin-impedance recording across dermatome sites. This is a further reason for the three test-sites recommendation for any given patient with the median value to be used in subsequent analysis.

The DDIM system is a static technique but demonstrates the extent of the skin abnormality at a level not previously documented in Fabry disease. The diagnostic potential of skin impedance in Fabry has not been explored but since the technique could potentially provide the busy clinician with a screening instrument for Fabry, further studies are indicated.

## Conclusion

Difference in skin-impedance between control and Fabry patients on ERT, and a non-significant difference between Fabry patients on ERT and Fabry ERT-naïve patients as assessed by DDIM system were demonstrated. To capture an optimum skin-moisture (sweating) with a minimum recording and also for data analysis ease, a median recording of R100 outcome measure over three test-sites on the right side of the body, Site 1 (Face; V1), Site 7 (Neck; T2) and Site 17 (Hand; radial) is recommended. Skin-impedance outcome measures failed to detect improvement in skin-moisture levels after two days interval testing in Fabry patients before and after ERT infusion. This may be due to the fact that unlike TST and QSART, the DDIM system assesses the base level of skin-moisture and not an induced sweating.

## Competing interests

The authors have no personal vested, economic, or capital interest in or relation with the Petite System other than its use in this study. Markus Ries MD, PhD, MHSc, FCP is an employee of Shire Human Genetic Therapies. The views and opinions expressed in this work do not necessarily reflect those of Shire Human Genetic Therapies.

## Authors' contributions

SNG participated in the design of the study, carried out patient testing and wrote the first draft of the manuscript. MR helped design the study, test the patients, and write the manuscript. GJM interpreted the GALA assays. JMQ carried out GALA assays. ROB helped write the manuscript. JRL helped with writing the manuscript and the statistical analysis. RS helped design and supervise the study and write the manuscript. DFM initiated the study, helped with its design, statistical analysis and participated in the writing of the manuscript. All authors read and approved the final manuscript.

## Pre-publication history

The pre-publication history for this paper can be accessed here:


